# Estimation of Heart Rate Recovery after Stair Climbing Using a Wrist-Worn Device

**DOI:** 10.3390/s19092113

**Published:** 2019-05-07

**Authors:** Daivaras Sokas, Andrius Petrėnas, Saulius Daukantas, Andrius Rapalis, Birutė Paliakaitė, Vaidotas Marozas

**Affiliations:** Biomedical Engineering Institute, Kaunas University of Technology, 51423 Kaunas, Lithuania; daivaras.sokas@ktu.lt (D.S.); saulius.daukantas@ktu.lt (S.D.); andrius.rapalis@ktu.lt (A.R.); birute.paliakaite@ktu.lt (B.P.); vaidotas.marozas@ktu.lt (V.M.)

**Keywords:** smart wristband, photoplethysmography, barometric pressure, altitude, physical activity, heart rate response, activities of daily living, long-term monitoring

## Abstract

Heart rate recovery (HRR) after physical exercise is a convenient method to assess cardiovascular autonomic function. Since stair climbing is a common daily activity, usually followed by a slow walking or rest, this type of activity can be considered as an alternative HRR test. The present study explores the feasibility to estimate HRR parameters after stair climbing using a wrist-worn device with embedded photoplethysmography and barometric pressure sensors. A custom-made wrist-worn device, capable of acquiring heart rate and altitude, was used to estimate the time-constant of exponential decay τ, the short-term time constant S, and the decay of heart rate in 1 min D. Fifty-four healthy volunteers were instructed to climb the stairs at three different climbing rates. When compared to the reference electrocardiogram, the absolute and percentage errors were found to be ≤ 21.0 s (≤ 52.7%) for τ, ≤ 0.14 (≤ 19.2%) for S, and ≤ 7.16 bpm (≤ 20.7%) for D in 75% of recovery phases available for analysis. The proposed approach to monitoring HRR parameters in an unobtrusive way may complement information provided by personal health monitoring devices (e.g., weight loss, physical activity), as well as have clinical relevance when evaluating the efficiency of cardiac rehabilitation program outside the clinical setting.

## 1. Introduction

Post-exercise heart rate recovery (HRR) has received great interest as a simple non-invasive approach to assess cardiovascular autonomic function [1,2,3]. Numerous studies show association of attenuated HRR with mortality and increased risk of cardiovascular disease, namely, coronary heart disease, myocardial infarction, and stroke [4,5,6,7,8,9]. Accordingly, it has been recently encouraged to include HRR assessment in routine clinical practice [3,10], e.g., as a fast and cheap alternative to ventilatory expired gas analysis [11]. Since HRR can be improved by cardiac rehabilitation [12,13,14] and even weight loss [15,16,17], monitoring of HRR trend over long run could be of interest when assessing the effectiveness of exercise training at home environment. Thus far, HRR is assessed using standardized tests which require specialized equipment and physician’s supervision, making such approach less convenient for use outside the clinical setting [18].

Advancements in wearable device technology have opened the possibility to estimate HRR in an unobtrusive way by acquiring heart rate from photoplethysmogram (PPG) signal. Some smartwatch manufacturers (e.g., Apple, Garmin) offer HRR estimation by allowing to measure how quickly heart rate returns to normal after intensive physical exercise. However, this principle is inconvenient since it requires manual device switching on into a special mode. Also, it is less acceptable for older individuals to whom interaction with wearable technologies is often complicated. These shortcomings remain to be solved, preferably by proposing solutions for unobtrusive HRR parameter monitoring without user intervention.

Given that stair climbing is a common daily activity, usually followed by a slow walking or rest, this type of activity can be considered as an alternative HRR test, performed in free-living conditions. Stairs climbed can be successfully detected using a barometric pressure sensor embedded in a wearable device. Barometric pressure captures altitude changes which is important information when detecting activities that involve body elevation [19,20,21,22].

This paper explores the feasibility to estimate HRR parameters after stair climbing using a wearable PPG-based device. A custom-made wrist-worn device, capable of acquiring raw PPG and altitude signals, as well as a consumer smart wristband, which provides heart rate, processed using proprietary algorithms, were thoroughly investigated. Parts of this research were presented in the conference paper [23], which reported results of a preliminary comparison study using a pilot database. This introductory paper has been substantially expanded, most importantly, by providing comprehensive investigation of HRR parameters on an enlarged database, composed of heart rate and altitude, recorded during stair climbing at different rates.

This paper is organized as follows. Section 2 describes a method for estimating HRR parameters after stair climbing using the PPG-based devices. Section 3 presents the results on a database recorded during stair climbing activities at different climbing rates. The paper ends with a discussion and conclusions.

## 2. Materials and Methods

### 2.1. Data Acquisition

Data collection took place indoors at Santaka Valley, Kaunas, Lithuania. A wrist-worn device, developed at Biomedical Engineering Institute of Kaunas University of Technology (Kaunas, Lithuania) was used for synchronously acquiring electrocardiogram (ECG), PPG, and barometric pressure. The analog front-end ADS1296R (Texas Instruments, Dallas, TX, USA) was used to acquire ECG at a sampling rate of 500 Hz, the analog front-end AFE4404 (Texas Instruments, Dallas, TX, USA) to acquire PPG at 100 Hz, and the sensor MS5611-01BA03 (TE Connectivity Ltd., Schaffhausen, Switzerland) to acquire barometric pressure at 50 Hz with an altitude resolution of 10 cm. Heart rate from the PPG signal was determined using a peak detector similar to the one described in [24]. For comparison, heart rate and climbed floors were obtained using a smart wristband Fitbit Charge 2 (Fitbit, San Francisco, CA, USA). It should be noted, that the consumer smart wristband does not provide access to the raw PPG signal, but rather heart rate at intervals of five seconds or longer, depending on the signal quality.

In fact, consumer wrist-worn devices (smart wristbands, smartwatches) estimate pulse rate assuming that a time interval between the adjacent pulse peaks in the PPG corresponds to the time interval between successive heart contractions. PPG-derived heart rate parameters show sufficient accuracy compared to the reference ECG [25,26,27] hence we use “heart rate” as a term to refer to pulse rate further in the paper. Heart rate series is denoted by *x*, where subscript *c* refers to the custom-made wrist-worn device, *f* to the consumer smart wristband, and *r* to the reference ECG.

### 2.2. Study Population

**Figure 1:**
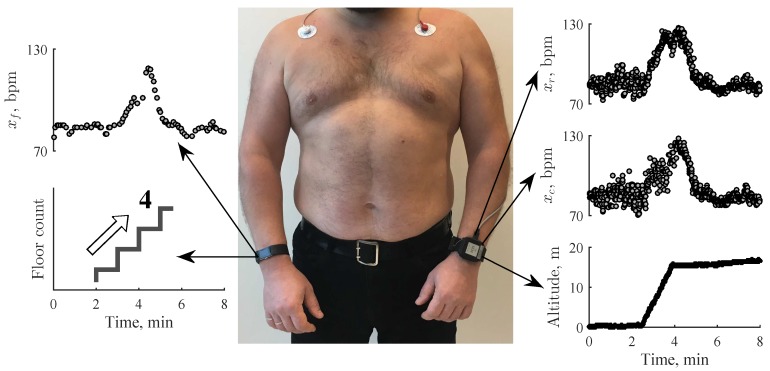
A custom-made wrist-worn device capable of acquiring reference electrocardiogram (ECG) ($x_{r}$), photoplethysmogram (PPG) ($x_{c}$), and barometric pressure (left arm). For comparison, heart rate ($x_{f}$) was synchronously obtained using a consumer smart wristband Fitbit Charge 2 (right arm).

**Figure 2:**
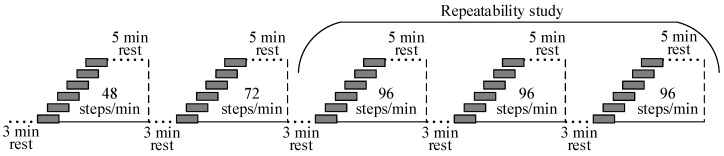
Protocol for acquisition of heart rate and altitude during standardized stair climbing activities.

Fifty-four healthy volunteers (18 women), 25.5 ± 8.1 years old (range 18 to 50 years), with a height of 177.4 ± 8.5 cm, weight of 73.7 ± 14.4 kg, and body mass index of 23.3 ± 3.9 kg/m${}^{2}$ participated in the study. All participants met the following criteria: (i) age ≥ 18 years, (ii) without documented cardiovascular disease, (iii) not taking β-blockers or calcium channel antagonists. The participants wore the wrist-worn device on the left wrist and Fitbit Charge 2 on the right wrist. A modified bipolar lead I, which is the voltage between the electrodes placed on the right and the left upper side of the chest, was used for acquiring reference heart rate (Figure 1). In accordance with the protocol of the YMCA bench step test for cardiovascular fitness [28], participants were asked to climb four floors of 96 stairs in total at different climbing rate, namely, 48, 72, and 96 steps per minute (Figure 2). To study HRR parameter repeatability, the latter activity was repeated three times. Steady stepping rate was ensured by an electronic metronome. The participants had to rest in a standing position for five minutes after each activity, slowly descend the stairs and rest three minutes before the next activity. The study was conducted in accordance to the ethical principles of the Declaration of Helsinki. Identifiable information was removed from the collected data to ensure participant anonymity.

### 2.3. Detection of Stair Climbing Activity

**Figure 3:**
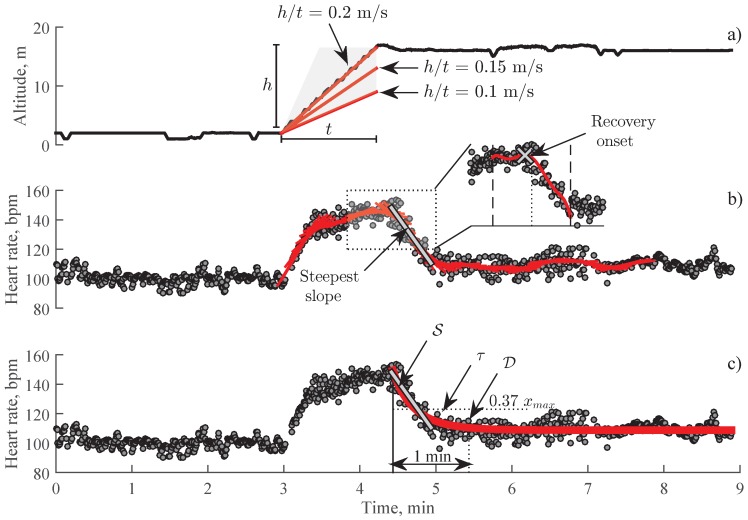
Estimation of heart rate recovery (HRR) parameters in heart rate series with a stair climbing activity: (**a**) altitude with the onset of stair climbing at 3 min, (**b**) detection of the steepest falling slope in a synchronously recorded heart rate series where the peak in the fitted polynomial corresponds to the recovery onset, (**c**) estimation of HRR parameters.

Barometric pressure is related to the elevation of a wrist-worn device above sea level. Since only the relative change in altitude is informative when detecting stair climbing, barometric pressure was calibrated by setting the ground floor to 0 m altitude. An activity of stair climbing is determined as a steady increase of altitude for ≥20 s at a minimum vertical speed of 0.1 m/s (Figure 3a). These thresholds were chosen empirically based on commonly observed stair climbing rates, as well as their effect on increase in heart rate, sufficient to induce noticeable heart rate decay.

### 2.4. Detection of Recovery Onset

Detection of recovery onset in heart rate series is only performed when an activity of stair climbing is detected in the altitude signal. Our observations show that the duration of full recovery depends on the climbing rate and usually lasts no longer than 5 min, which is consistent with the duration of HRR phase, observed when performing submaximal and maximal standardized tests [18].

When a stair climbing activity is detected, a search for the recovery onset is performed by fitting a linear polynomial to heart rate series in a sliding window of 1 min. Then, the time interval with the steepest falling slope is chosen as a suspected recovery interval (Figure 3b). The heart rate series 25 s before and after the beginning of the steepest falling curve is extracted for fitting 6th order polynomial function where the maximal value determines the recovery onset (Figure 3b).

### 2.5. Estimation of Heart Rate Recovery

With the onset of physical activity, heart rate starts to increase due to parasympathetic withdrawal and sympathetic activation, whereas decays towards its pre-exercise level after the end of physical activity due to parasympathetic reactivation and sympathetic withdrawal. Recovery period can further be divided into the fast and slow phases with different physiological meanings. The fast decrease in heart rate that occurs immediately after the end of physical activity is entirely due to increase in parasympathetic activity, driven by the deactivation of central cardiovascular control mechanism in the brain and the abolished feedback from muscle mechanoreceptors. The subsequent slow decrease in heart rate results from coordinated parasympathetic-sympathetic interaction, mediated by the reduced feedback from muscle metaboreceptors and the adjustments in thermoregulation [3].

Heart rate normally recovers in an exponential manner, thus can be approximated by a mono-exponential model [29,30],

(1) $$x_{m}\left(t\right)=x_{0}+x_{\Delta }e^{-\frac{t}{\tau }},$$

where $x_{0}$ is the heart rate at the end of recovery phase, $x_{\Delta }$ is the difference between the heart rate at the beginning and the end of recovery phase, and τ is the time-constant of an exponential decay (see Figure 3c). The time-constant of the exponential decay τ is associated with parasympathetic reactivation and sympathetic withdrawal [29].

**Figure 4:**
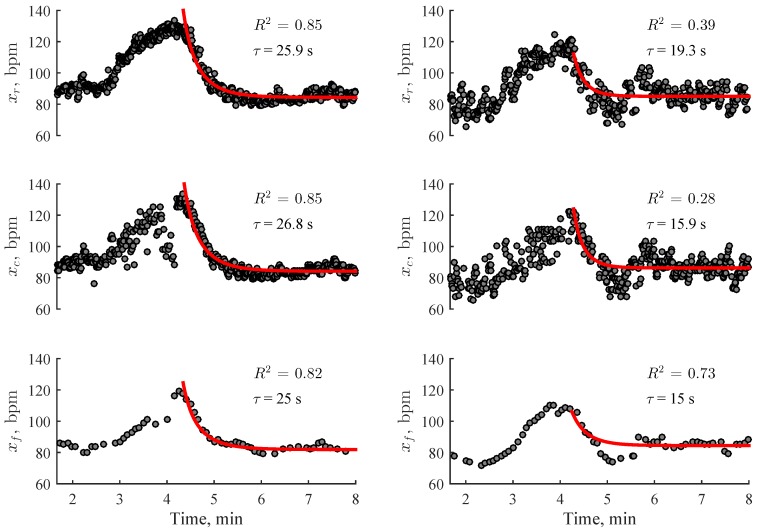
Examples of exponential fitting to heart rate series after the stair climbing activity. Slower recovery results in larger $R^{2}$ values (left column), whereas faster recovery increases heart rate variability in the slow recovery phase, and thus, reduces $R^{2}$ to such an extent that fitting is considered no longer reliable (right column). Note that $R^{2}$ is often larger for heart rate series acquired using smart wristband Fitbit Charge 2 due to processed heart rate series using proprietary algorithms, which artificially reduce variability.

The exponential model is fitted in a time interval of 5 min, starting at the recovery onset. We assume that heart rate achieves at least 95% of full recovery within 5 min after the stair climbing activity. Based on this assumption, τ > 100 s was considered as a fitting error and therefore excluded from further analysis. The quality of exponential fitting is determined via the coefficient of determination $R^{2}$. Fitting is considered acceptable when $R^{2}$ exceeds the empirically chosen fixed threshold of 0.5. Differently from other studies where larger $R^{2}$ thresholds were used to determine the acceptable fitting quality [30], the decision for choosing lower threshold was based on the specialty of the stair climbing activity, which is less intensive than maximal or submaximal trendmill/ergometer exercise. Since heart recovers faster after stair climbing, manifestation of heart rate variability in the slow recovery phase may reduce $R^{2}$ considerably, despite exponential curve matches well with decay tendencies (Figure 4).

The rapid heart rate decay immediately after the recovery onset, which reflects parasympathetic reactivation, is characterized by the short-term time constant S [31]. S is found by fitting a linear polynomial to the logarithm of heart rate. Then, S is the negative inverse of the slope of the resulting line, expressed as −1/slope. To ensure better reproducibility, S is estimated within the first second after the recovery onset in a sliding window of 30 s and the lowest value is selected [32].

Decay of heart rate in 1 min, here denoted as D, is a well-established parameter that reflects parasympathetic reactivation [18]. D is estimated by finding the difference between the heart rate at recovery onset and the heart rate after 1 min (see Figure 3c).

### 2.6. Performance Evaluation

Bland–Altman plots are used to evaluate and display the agreement between the HRR parameters estimated using the reference and the PPG-based devices, i.e., the wrist-worn device and the smart wristband.

HRR parameter variation in three consecutive stair climbing activities (repeatability study), performed on the same participant under nearly identical conditions, is assessed using the repeatability coefficient which is defined by:

(2) $$r=1.96\cdot \sqrt{2}\cdot \sigma ,$$

where σ is an estimate of the within-participant standard deviation, obtained by fitting a one-way analysis of variance (ANOVA) model to the repeatedly acquired HRR parameters [33]. It is expected that the difference between the estimated parameters on the same participant will be less than *r* in 95% of occasions.

## 3. Results

Table 1 presents quality analysis of heart rate series acquired during stair climbing. Regardless of the device used to obtain the data, about 10% of heart rate series were corrupted by artefacts or had segments of lost data. Much larger number of heart rate series was excluded due to unacceptable fitting quality ($R^{2}$ < 0.5), except for the smart wristband. Visual inspection of such excluded recovery phases revealed that about 20% of them did not met the quality criterion despite the fitted curve coincided well with the decay tendencies.

Analysis of $R^{2}$ values for the reference ECG show that a number of heart rate series that satisfy the quality criterion increases for higher climbing rates, which a consequence of reduced heart rate variability during the slow recovery phase. As expected, the lowest number of excluded recovery phases due to $R^{2}$ < 0.5, as well as the best average fit ($R^{2}$ = 0.78–0.84) are obtained for heart rate series from the smart wristband, which can be explained by artificially reduced heart rate variability due to embedded heart rate averaging. Similar $R^{2}$ values are obtained for the reference ECG and the wrist-worn device, being 0.64–0.68 and 0.65–0.67, respectively.

**Figure 5:**
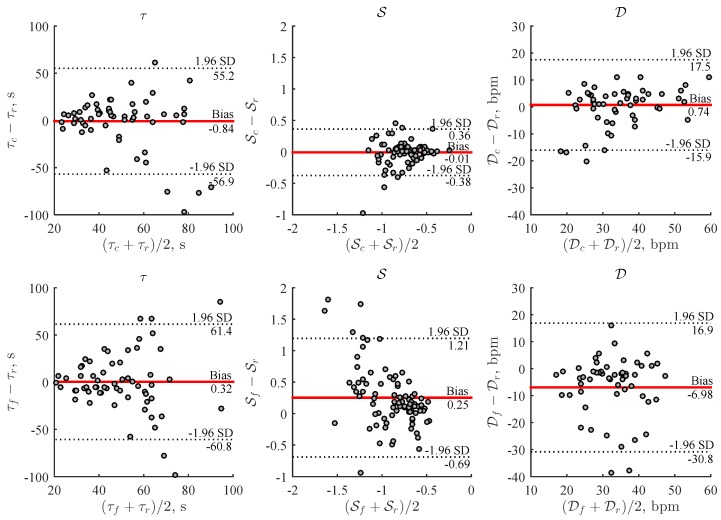
Comparison of HRR parameters estimated using the wrist-worn device and smart wristband with respect to the reference ECG at a stair climbing rate of 96 steps/min (data of all three repeated stair climbing activities were included).

Figure 5 shows the Bland–Altman plots of HRR parameters estimated from the reference ECG and PPG-based devices. The absolute and percentage errors were found to be ≤21.0 s (≤52.7%) for τ, ≤0.14 (≤19.2%) for S, and ≤7.16 bpm (≤20.7%) for D in 75% of recovery phases available for analysis. When comparing PPG-based devices, the parameters S and D show 2.4 and 1.4 times narrower limits of agreement for the wrist-worn device.

**Figure 6:**
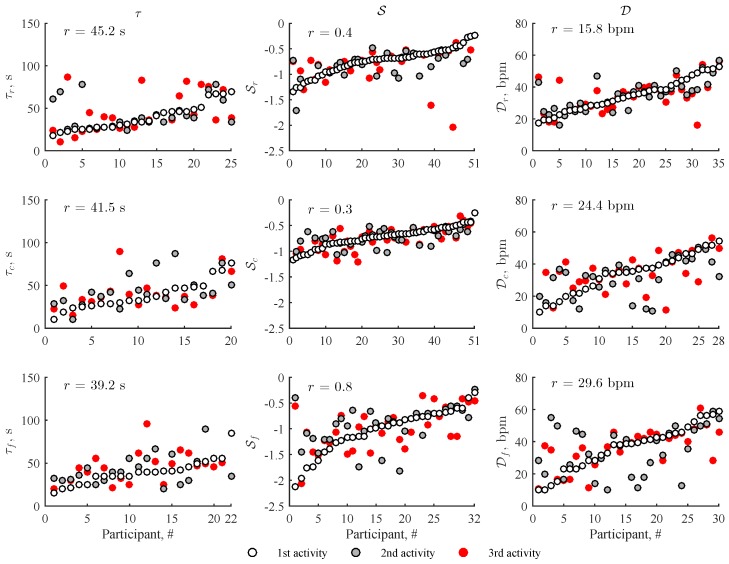
Repeatability of HRR parameters obtained for three consecutive stair climbing activities at a rate of 96 steps/min. The HRR parameter values are sorted with respect to the first stair climbing activity. Note that only those participants whose all three repeated stair climbing activities met the quality criterion of $R^{2}$ > 0.5 and had no corrupted segments were included in the repeatability study.

HRR parameter repeatability was assessed only for those participants whose all three repeated stair climbing activities met the quality criterion of $R^{2}$ > 0.5 (Figure 6). HRR parameter values exhibit substantial variability for large part of the participants despite that stair climbing activity was performed under nearly identical conditions. This finding suggests that physiological factors, such as previously experienced physical activity, may play an important role on HRR. Both PPG-based devices show similar repeatability for τ and D, with the exception of S, for which the wrist-worn device is superior. The smart wristband causes large errors since S is sensitive to the slope of the HRR curve, which is affected by the heart rate averaging and shifted onset of recovery phase.

**Figure 7:**
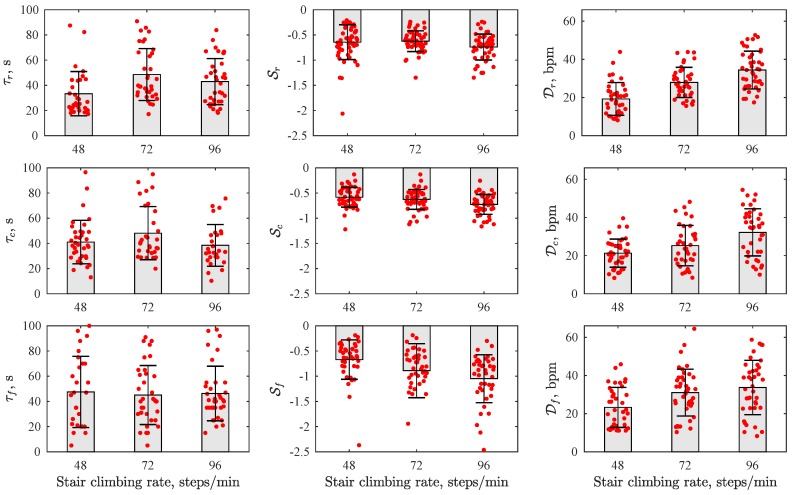
HRR parameters estimated at different stair climbing rates.

Figure 7 displays HRR parameters estimated at different stair climbing rates. The results show obvious dependence of parameter D on a stair climbing rate, i.e., the difference between the heart rate at recovery onset and that after 1 min increases as climbing intensity increases. Similarly, the steepness of the recovery slope S tends to slightly increase for higher climbing rates.

## 4. Discussion

The aim of the study was to investigate the feasibility to estimate HRR parameters after stair climbing using a wrist-worn PPG-based device. Thus far, only straightforward information, such as time spent in a specific heart rate zone, is being provided for self-monitoring and performance feedback by smart wristband manufacturers. With this study, we seek to take further step towards collecting more comprehensive information about the health status. The capability to monitor HRR over time can be relevant both commercially and from the clinical viewpoint. That is, linking HRR trend changes to other information, provided by personal health monitoring devices, may spark interest from smart wristband manufacturers. Also, HRR monitoring may facilitate evaluation of the efficiency of cardiac rehabilitation at home environment.

Considering the application of the proposed approach to HRR monitoring in activities of daily living, both altitude and heart rate series should preferably be used to detect the recovery onset. Presumably, the subject will not rest immediately after stair climbing therefore the end of the activity would not necessarily correspond to the maximum heart rate. Heart rate may increase for some time, especially after low intensity climbing thus it is not enough to rely on altitude alone and heart rate series should be inspected to find the precise point of the recovery onset. In addition, unlike the treadmill/ergometer exercise, which elevates the heart rate up to submaximal or maximal level at which heart rate variability disappears, stair climbing is often not challenging enough, making the precise detection of the recovery onset in a variable heart rate complicated. A polynomial fitting was chosen to obtain a smooth curve in which the maximum value corresponds to the recovery onset. The goal was to keep the order of the polynomial function as low as possible since higher order model may have too many inflection points and conform to the tendencies of heart rate variability.

Post-exercise heart recovery can be divided into fast and slow recovery phases [31,34,35]. The fast recovery phase usually takes about one minute, and is followed by the slow phase which continuous until heart rate reaches the resting value. A common approach to characterize the fast recovery phase is to take the difference between the heart rate at recovery onset and the heart rate at 30 s, 1 min, or 2 min. In this study, the reference point at 1 min (parameter D) was chosen taking into account the elucidated clinical significance. Numerous studies show that D ≤ 12 bpm is an independent predictor of both cardiovascular and all-cause mortality [4,5,36,37]. Differently from D, which reflects a single heart rate value, the short-term time constant S addresses the slope of the fast recovery phase, and thus, may represent different HRR characteristics. Meanwhile, the exponential constant τ covers both fast and slow phases [3]. Decay of heart rate after 5 min of recovery onset is another widely studied parameter which addresses both fast and slow phases, however, was not included in the study due to its obvious dependance on D [8,38].

Study shows large within-subject parameter variation, especially τ, even when successive stair climbing activities were performed under nearly identical conditions. This finding is in agreement with previous reports that τ is least reproducible, particularly after low intensity exercise [39,40]. Such factors as precise detection of recovery onset, duration of fast recovery phase, heart rate errors, and heart rate variability during the slow recovery phase might influence the fitting of the exponential model [18,29]. Considering that HRR parameters often show higher reproducibility following maximal exercise [41,42], within-subject parameter variation could potentially be reduced at higher climbing rates. Since this study had a purpose to replicate ordinary stair climbing, unrealistically high climbing rates, which could reflect submaximal exercise, were not included in the study protocol. Large parameter variation can also be ascribed to external factors such as previously experienced physical activity. This cannot be controlled in free-living activities hence should be accepted as an integral part of the proposed approach to HRR assessment. While a single measurement of HRR would not be enough to capture effect of a certain exposure (e.g., physical activity, weight loss, etc.), it remains to be shown whether within-subject variation is acceptable to detect trend changes over long run.

Most studies have utilized resting postures, namely, supine, seated, standing, after physical exercise, however, complete rest is rare in activities of daily living. Changes in posture (e.g., while removing clothes and shoes), as well as low intensity activity (e.g., slow walking) can often be expected immediately after stair climbing. While posture changes may cause substantial alterations in autonomic function [43], the effect of walking after physical activity is unclear. One of the few studies demonstrated high reproducibility of HRR parameters estimated during walking after exhaustive running in athletes [41]. However, the effect of the aforementioned factors on parameter reproducibility after stair climbing has still to be addressed.

In principal, HRR can be assessed after fast walking [44]. Since stair climbing requires to lift the body mass against gravity, it is more energy demanding activity, and is therefore a better option to cause a substantial increase in heart rate. In addition, stair climbing was found to be correlated with leg power impairment in mobility-limited older adults [45] and motor recovery after brain stroke [46]. The latter information can only be obtained using a specialized equipment, which greatly limits large-scale applicability, thus the possibility to characterize the properties of stair climbing using wearable devices deserves further exploration.

High user compliance is crucial to assure gap-free long-term monitoring, making the selection of device placement site of great importance. Various placement sites have been considered, namely, trunk, waist, wrist, ankle, pelvis, etc. Physical activity using a wrist-worn device can be assessed solely via accelerometer in exchange to lower accuracy compared to more motion-resistant placement sites. Interestingly, by including barometric pressure and by placing the signal acquisition modules on the wrist, chest and ankles, the accuracy of classifying various activities of daily living was the highest when the device was placed on the wrist [21]. When detecting stair ascent and descent, an improvement of 20% in the classification accuracy was achieved [21], emphasizing the importance of barometric pressure sensor.

Consumer smart wristbands are optimized to save battery life, therefore prefer low PPG sampling rate (e.g., 25 Hz) and generally do not provide beat-to-beat heart rate. Unsurprisingly, study findings show that heart rate series from the smart wristband leads to considerable HRR parameter estimation errors, which is in agreement with the results of our previous work [44]. Regardless of this, the availability of miscellaneous data (heart rate, steps, climbed floors, etc.) is particularly attractive, therefore, can potentially be used for estimating HRR using third party applications. Since only a small fraction of manufacturers provide access to their data, the smart wristband of only one manufacturer has been investigated. Other manufacturers may use different signal processing algorithms, thereby liming the generalizability of the results to different smart wristbands.

The current study has limitations. To meet the requirements of repeatability study, which refers to the repeated measurements made on the same subject under identical conditions during which the underlying HRR parameter can be considered as constant, we separated three consecutive stair climbing activities by only 8 min rest periods. Ideally, variability in HRR parameter measurements on the same subject should be ascribed to errors due to the measurement process itself. Nevertheless, the separation of consecutive stair climbing activities by 8 min may influence HRR of the upcoming stair climbing activity, and thus induce HRR parameter variation due to underlying physiological changes. This reasoning is based on the known alteration in autonomic balance lasting up to 24 h following low-intensity physical exercise [47].

**Figure 8:**
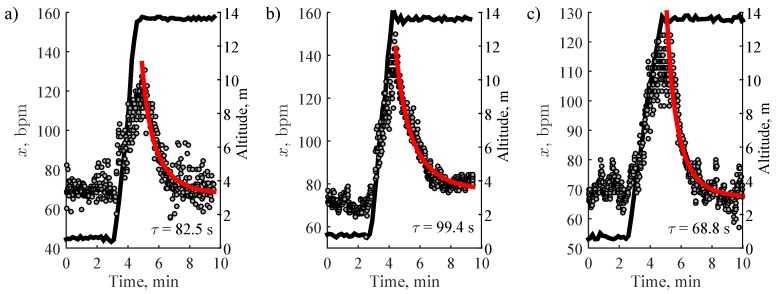
Stair climbing activities from the elderly individuals, instructed to safely ascend the stairs at their comfortable speed: (**a**) 74-year-old female, average stair climbing rate—61 steps/min, (**b)** 72-year-old female, average stair climbing rate—65 steps/min, (**c**) 74-year-old female, average stair climbing rate—42 steps/min. The black line stands for altitude.

The relatively young cohort of healthy participants, recruited to perform standardized stair climbing, certainly limits the generalization of the study findings to other population groups. Elderly individuals may prefer considerably different climbing patterns with different speed and rest intervals. Since our study cohort was relatively young and obviously in a superior shape than most elderly individuals, the lowest stepping rate of 48 steps per minute was selected as the minimal which can still induce noticeable heart rate response to physical activity. Some elderly individuals may climb the stairs at lower stepping rates, however, even that can be enough to cause considerable heart response. This can be supported by the examples of stair climbing activities of elderly individuals (Figure 8), which demonstrate that even low climbing rate (42–65 steps/min) induces substantial increase in heart rate.

Finally, since the current study included only healthy participants, this limits the extrapolation of the results to those with autonomic dysfunction or cardiovascular disease.

## 5. Conclusions

This study demonstrates the feasibility to estimate heart rate recovery after stair climbing using the wrist-worn device with embedded photoplethysmography and barometric pressure sensors. According to the study results, the short-term time constant and the decay of heart rate in 1 min after the recovery onset are the most accurate and repeatable parameters, whereas the time-constant of exponential decay is least reliable. The proposed approach to monitoring HRR is expected to be valuable when combining with other information provided by personal health monitoring devices (e.g., weight loss, physical activity), as well as have relevance when evaluating the efficiency of cardiac rehabilitation program outside the clinical setting.

## Figures and Tables

**Table 1:** Quality analysis of heart rate series acquired for a particular climbing rate. The results are given as the number of heart rate series that satisfy the above stated condition/the total number of heart rate series. $R^{2}$ values that met the quality criterion of $R^{2}$ > 0.5 are given as mean ± standard deviation.

**Reference ECG**					
Stair climbing rate	$R^{2}$ > 0.5	$R^{2}$ < 0.5	τ > 100 s	Corrupted	$R^{2}$ ($R^{2}$ > 0.5)
48 steps/min	33/54 (61.1%)	11/54 (20.4%)	6/54 (11.1%)	4/54 (7.4%)	0.64 ± 0.12
72 steps/min	38/54 (70.3%)	7/54 (13.0%)	4/54 (7.4%)	5/54 (9.3%)	0.64 ± 0.06
96 steps/min	112/162 (69.1%)	12/162 (7.4%)	17/162 (10.5%)	21/162 (13.0%)	0.68 ± 0.11
**Wrist-Worn Device**					
Stair climbing rate	$R^{2}$ > 0.5	$R^{2}$ < 0.5	τ > 100 s	Corrupted	$R^{2}$ ($R^{2}$ > 0.5)
48 steps/min	26/54 (48.1%)	16/54 (29.6%)	8/54 (14.8%)	4/54 (7.4%)	0.65 ± 0.09
72 steps/min	32/54 (59.3%)	14/54 (25.9%)	4/54 (7.4%)	4/54 (7.4%)	0.67 ± 0.16
96 steps/min	86/162 (53.1%)	45/162 (27.8%)	16/162 (9.9%)	15/162 (9.3%)	0.66 ± 0.12
**Smart Wristband**					
Stair climbing rate	$R^{2}$ > 0.5	$R^{2}$ < 0.5	τ > 100 s	Corrupted	$R^{2}$ ($R^{2}$ > 0.5)
48 steps/min	34/54 (62.9%)	8/54 (14.8%)	5/54 (9.3%)	7/54 (13.0%)	0.78 ± 0.13
72 steps/min	35/54 (64.8%)	3/54 (5.6%)	6/54 (11.1%)	10/54 (18.5%)	0.79 ± 0.11
96 steps/min	104/162 (64.2%)	33/162 (20.4%)	16/162 (9.9%)	9/162 (5.6%)	0.84 ± 0.11

## Sample Availability

The data samples acquired during stair climbing using a wrist-worn device are available from the corresponding author on request.

## Abbreviations

The following abbreviations are used in this manuscript:

HRR	Heart rate recovery
PPG	Photoplethysmogram
ECG	Electrocardiogram
